# Efficient EEG channel-and-frequency-band selection for epileptic seizure classification using multi-objective optimization

**DOI:** 10.3389/fneur.2026.1831912

**Published:** 2026-04-29

**Authors:** Wenjie Chen, Xinqi Lei, Hainan Guo, Li Zhuang

**Affiliations:** 1 The School of Information Management, Central China Normal University, Wuhan, China; 2 The College of Management, Shenzhen University, Shenzhen, China; 3 The School of Cyber Science and Engineering, Southeast University, Nanjing, China

**Keywords:** channel-and-frequency-band selection, EEG signals, epileptic seizure classification, machine learning, multi-objective optimization

## Abstract

**Objective:**

With the rapid development of wearable electroencephalogram (EEG) devices, the epileptic seizure classification system is required to deliver reliable performance under real-time and resource-constrained conditions. To this end, this study aims to reduce EEG signal acquisition and processing costs while maintaining seizure classification performance in order to facilitate the clinical deployment of intelligent EEG analysis systems.

**Methods:**

We jointly optimize the number of EEG channels and frequency bands, with the goals of maximizing classification performance while minimizing signal acquisition and computational costs. The proposed optimization problem is solved by the structure-aware non-dominated sorting genetic algorithm II (SA-NSGA-II). The random forest method is employed as the classifier for seizure classification. Experiments are conducted using the public CHB-MIT scalp EEG database.

**Results:**

Among the optimal channel-and-frequency-band configurations, channels P3-O1, P4-O2, and CZ-PZ are selected with high frequencies, indicating their high relevance for seizure classification. Furthermore, the gamma and alpha bands account for the largest two selection proportions, which suggests their key roles in optimal configurations. In addition, the proposed SA-NSGA-II method demonstrates effective performance in the EEG channel-and-frequency-band selection.

**Conclusion:**

The proposed framework effectively balances classification performance with EEG acquisition and computational costs. By jointly selecting channels and frequency bands, our method provides an easy-to-implement solution for resource-efficient seizure classification in real-time EEG monitoring.

## 1 Introduction

Epilepsy is a common and highly heterogeneous neurological disorder. EEG-based automatic seizure classification plays an important role in clinical diagnosis support, long-term monitoring, personalized treatment (1). With the development of wearable EEG devices and bedside monitoring systems, seizure classification methods must not only deliver reliable classification performance, but also meet the demands of real-time processing and operation under resource-constrained conditions (2, 3). In practice, multi-channel EEG acquisition and complex signal processing often lead to substantial computational, energy, and bandwidth overhead (4). Therefore, reducing the cost of signal acquisition and processing while maintaining classification performance has become a critical challenge for bringing intelligent EEG analysis into clinical practice.

Most existing works focus on using full-channel and full-band EEG signals for feature extraction or building complex machine learning models to improve seizure classification accuracy (5, 6). These approaches have achieved strong classification performance in offline experiments. However, full-channel and full-band EEG signals hold high-dimensional spatial and spectral information. This means that these methods require continuous multi-channel signal acquisition and full-spectrum processing, which significantly increases system complexity and energy consumption in real-time applications. Optimizing classification performance without explicitly modeling the costs associated with channel acquisition and frequency band processing makes them difficult to deploy in resource-limited settings such as wearable or long-term monitoring systems.

From a neurophysiological perspective, epileptic EEG signals exhibit notable heterogeneity across both spatial and spectral dimensions. Seizure-related activity often emerges selectively in specific cortical regions and their corresponding frequency bands, rather than being evenly distributed across all channels or the entire spectrum (7). Some studies have attempted to reduce EEG acquisition costs through channel selection (8, 9). Channel-level features can offer a compact representation of global brain activity. However, they inherently aggregate contributions from multiple frequency components and potentially mask the frequency-specific pathological information that is crucial for seizure classification. From a modeling perspective, relying only on channel selection implicitly assumes that all frequency components carry equal informational value and should be processed uniformly. This not only limits the ability to capture spectral redundancy but also prevents explicit modeling and optimization of band-level computational costs.

Motivated by these facts, this paper aims to reduce both acquisition and processing costs while preserving seizure classification performance. We propose a multi-objective optimization framework that jointly models channel-and-frequency-band selection. By explicitly introducing decision variables for channel-and-frequency-band selection and characterizing the structural constraints between them, the proposed method enables EEG representation at both spatial and spectral levels to achieve a balance between classification performance and system efficiency. Our approach not only aligns more closely with the physiological characteristics of epileptic EEG, but also offers a practically promising solution for real-time seizure classification in resource-constrained environments.

To effectively solve the multi-objective optimization problem, we further introduce a structure-aware non-dominated sorting genetic algorithm II (SA-NSGA-II). Considering the structural dependencies between channels and frequency bands, the SA-NSGA-II algorithm incorporates structure-preserving initialization (SPI) and structure-guided local search (SGLS) to efficiently identify optimal channel and band combinations. The experiment results provide the statistical analysis of the importance of different channels and frequency bands. They also reveal the superiority of SA-NSGA-II compared to the standard optimization algorithm.

## 2 Materials and methods

### 2.1 EEG database introduction

#### 2.1.1 CHB-MIT scalp EEG database

Data for this research are sourced from the CHB-MIT Scalp EEG Database, a standard benchmark in seizure classification (10). Collected at Boston Children's Hospital, this dataset includes EEG recordings from 23 pediatric patients with intractable seizures. The data are categorized into seizure and non-seizure segments where the seizure segment has at least one seizure event and the non-seizure segment records the interictal state. The EEG signals are obtained at a sampling rate of 256 Hz with 16-bit resolution using a bipolar montage. Each channel records the difference between two specific electrode sites. Each patient's data has at least 22 common channels (e.g., FP1-F7, F7-T7, etc.i), and the electrode configurations follow the standardized International 10–20 system.

#### 2.1.2 Data pre-processing

This paper uses the EEG recordings from 23 patients with 22 common channels for seizure classification. First, the raw EEG signals are pre-processed to remove the noise. A finite impulse response band-pass filter with a frequency range of 0.5–50 Hz is applied. This range covers five major EEG frequency bands commonly associated with epileptic activity, including the δ (0.5–4 Hz), θ (4–8 Hz), α (8–13 Hz), β (13–30 Hz), and γ (>30 Hz) bands to provide reliable signals for subsequent frequency-band analysis. Then, we downsample the EEG signals from 256 Hz to 128 Hz because it is demonstrated that seizure prediction performance does not differ significantly between the sampling rates of 256 Hz and 128 Hz while the sampling rate of 128 Hz can significantly reduce the computational cost (11). In addition, since the amplitude of the EEG signals is around ±10^−4^, the signals are multiplied by 10^4^ for amplitude normalization to facilitate model training.

In this study, the classification of preictal and interictal EEG signals is the main task for seizure classification. The 10-min EEG segment preceding each seizure is defined as the preictal period and labeled as 1, while 10-min EEG segment extracted from non-seizure recordings is defined as the interictal period and labeled as 0 (9, 12). Each 10-min EEG segment is divided into 8-s segments using a sliding window with a step size of 1 s, and each segment is treated as an independent sample. For each patient, we randomly select two preictal segments and two interictal segments. Accordingly, we have 1,186 preictal samples and 1,186 interictal samples for each patient.

### 2.2 Epileptic seizure classification

#### 2.2.1 Feature extraction

For EEG signals sampled at 128 Hz, a four-level discrete wavelet transform is implemented to decompose and reconstruct the signals into five clinical frequency bands: the approximation coefficients cA4 (0–4 Hz) corresponding to the δ band, and the detail coefficients cD4 (4–8 Hz), cD3 (8–16 Hz), cD2 (16–32 Hz), and cD1 (32–64 Hz) corresponding to the θ, α, β, and γ bands, respectively (13). Subsequently, for each 8-s epoch per channel, the mean energy of the pre-processed channel signal is extracted as the channel-level feature, while the variances of the five frequency bands are computed as band-level features. By concatenating the mean energy from 22 channels and the variance features of the five frequency bands channel, a 132-dimensional feature vector is generated for each 8-s segment.

#### 2.2.2 Classification model and training protocol

For model construction, a previous study compared the performance of various machine learning methods in seizure classification, including traditional machine learning algorithms such as random forest and gradient boosting, as well as deep learning models such as long short-term memory networks and long recurrent convolutional networks (14). Results show that the random forest model exhibits high classification performance. Although deep learning models can also achieve good results, they require a large number of training samples and have high computational complexity.

Considering the strong requirements for computational efficiency in this study, we choose the random forest model as the base classifier for modeling. Samples are divided into the training set and the test set in an 4:1 ratio. Three-fold cross-validation is used for model selection. The hyperparameters of the random forest model are optimized using the random search method.

### 2.3 Channel-and-frequency-band joint optimization

#### 2.3.1 Multi-objective optimization problem formulation

po balance classification performance and resource consumption, the channel-and-frequency-band selection task is formulated as a multi-objective optimization framework. Specifically, the goal is to maximize the seizure classification accuracy while minimizing the number of selected EEG channels and the number of processed frequency bands. Let *C* and *K* represent the total number of channels and frequency bands, respectively. Binary decision variables *s*_*c*_∈{0, 1} are used to indicate whether the *c*-th channel is selected, and *z*_*c, k*_∈{0, 1} indicates whether the *k*-th frequency band of channel *c* is included. The optimization objectives are defined as follows:


$$\begin{array}{l} \begin{array}{l} \underset{s,z}{\max}\;f_{1}\left(s,z\right)=A\left(s,z\right)\\ \underset{s}{\min}\;f_{2}\left(s\right)=\overset{C}{\underset{c=1}{\sum }}s_{c}\\ \underset{z}{\min}\;f_{3}\left(z\right)=\overset{C}{\underset{c=1}{\sum }}\overset{K}{\underset{k=1}{\sum }}z_{c,k} \end{array} \end{array}$$

where A(s,z) represents the seizure classification accuracy under the selected channel-and-frequency-band configuration.

To ensure that frequency bands can only be selected when their corresponding channels are activated, a structural constraint is imposed: *z*_*c, k*_ ≤ *s*_*c*_, ∀*c*∈{1, ..., *C*} and ∀*k*∈{1, ..., *K*}. This model explicitly captures the trade-off between diagnostic performance and acquisition/processing burden for real-time and resource-constrained EEG monitoring scenarios.

#### 2.3.2 Structure-aware NSGA-II optimization algorithm

The non-dominated sorting genetic algorithm II (NSGA-II) has been widely used in the EEG channel selection problems (15). However, our problem involves the structural constraint between the channel selection and frequency-band selection. The conventional NSGA-II does not consider the structure dependence, which may generate infeasible configurations and reduce search efficiency. To address this issue, we develop a structure-aware extension of NSGA-II, referred to as SA-NSGA-II. Two structure-aware mechanisms, structure-preserving initialization (SPI) and structure-guided local search (SGLS), are incorporated into the evolutionary process.

SPI ensures that all initial individuals satisfy the predefined channel-and-frequency-band channel-and-frequency-band coupling constraints. Instead of independently selecting each solution is generated by first sampling a subset of channels, followed by assigning frequency-band selections based on the activated channels. If a channel is not selected, all its associated frequency-band variables are automatically set to zero. This initialization mechanism ensures that the search begins in the feasible configuration space.

SGLS is applied with a predefined probability after crossover and mutation to refine high-quality solutions. Three move operators are defined: (i) Channel-off move, where an active channel and all its associated frequency bands are deactivated; (ii) Channel-on move, where an inactive channel is activated, and one frequency band is randomly enabled; (iii) Band-fine-tuning move, where for an active channel, one frequency band is selectively deactivated or activated. After each move, the modified solution is re-evaluated and accepted only if it is not dominated by the original individual. This mechanism improves the solution quality while maintaining effective channel-and-frequency-band configurations. Except for these two modifications, the remaining components follow the standard NSGA-II framework (16).

## 3 Results

### 3.1 Multi-objective optimization results

**Figure 1:**
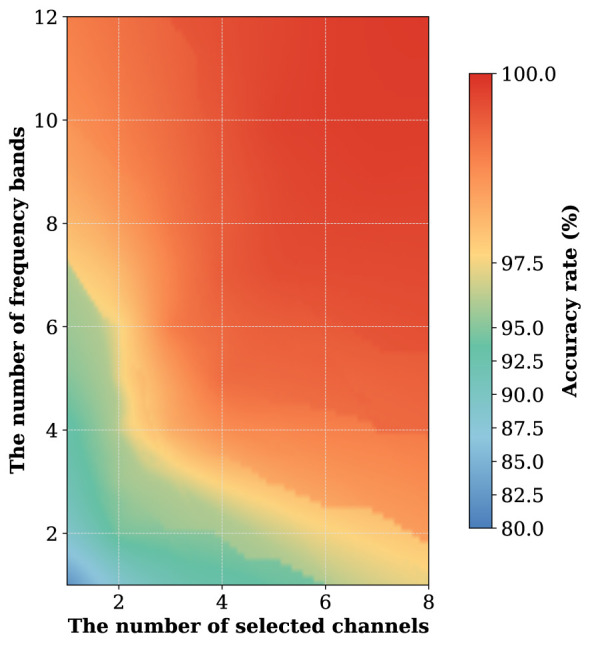
The spatial distribution of the optimal channel-and-frequency-band configurations.

For SA-NSGA-II, the population size is set to 50, the crossover probability to 0.9, and the mutation probability to 1/*N*, where *N* denotes the number of selected channels and frequency bands. The probability of the structure-guided local search operation is set to 0.15 to balance the search efficiency and solution quality. To evaluate the effectiveness of the proposed multi-objective optimization framework, we first analyze the distribution of the Pareto-optimal solutions across three dimensions: classification accuracy, the number of selected EEG channels, and the number of selected frequency bands. Figure 1 shows the spatial distribution of the optimal channel-and-frequency-band configurations. It is observed that classification performance improves as the number of channels and frequency bands increases. When only a small number of channels and frequency bands are used, the accuracy remains relatively low, indicating insufficient spatial and spectral information for reliable seizure classification. As the number of selected channels increases from 1 to approximately 4, and the number of frequency bands increases from 1 to around 6, the classification accuracy improves substantially. Beyond this range, the improvement becomes relatively moderate. This finding demonstrates diminishing returns when additional channels or frequency bands are incorporated. In particular, high classification accuracy (above 98%) can already be achieved using a limited number of channels and frequency bands. Therefore, by analyzing the threshold, the practitioner can identify the knee points that maintain acceptable diagnostic performance while achieving the most reasonable resource consumption.

To further guide clinical decision-making, we categorize the Pareto solutions into three distinct performance-cost sets. (i) High-performance set: This configuration prioritizes the maximum diagnostic reliability, achieving a seizure classification accuracy exceeding 99.5% by utilizing extensive channels and frequency bands. (ii) Balanced-efficiency set: This selection offers a strategic compromise, maintaining robust classification performance (>97.5%) while reducing the number of active channels and required bandwidth. (iii) Low-resource set: This profile prioritizes minimal resource consumption for long-term monitoring applications although classification accuracy is lower than 97.5%. We choose some representative configurations and summary them in Table 1. It is shown that increasing the number of selected channels or frequency bands can improve the classification accuracy. When we only select one channel and one frequency band, the lowest accuracy is 82.17%.

**Table 1:** Representative configurations in the Pareto-optimal set.

Category	Index	Accuracy (*%*)	Number of selected channels	Number of selected frequency bands
High-performance set	1	99.83	8	12
	2	99.70	5	10
	3	99.53	5	7
Balanced-efficiency set	1	99.41	4	9
	2	98.90	3	6
	3	97.98	2	7
Low-resource set	1	97.13	3	3
	2	92.88	1	4
	3	82.17	1	1

### 3.2 Channel-and-frequency-band selection analysis

This section aims to provide insight into which spatial regions and spectral components contribute most to seizure classification while maintaining low acquisition and processing costs. To this end, we analyze the distribution patterns of selected EEG channels and frequency bands across the Pareto-optimal solution set obtained by SA-NSGA-II. Specifically, we examine (i) the distribution of the selected EEG channels, (ii) the distribution of the selected frequency bands, and (iii) the joint utilization patterns of channel-and-frequency-band pairs.

**Figure 2:**
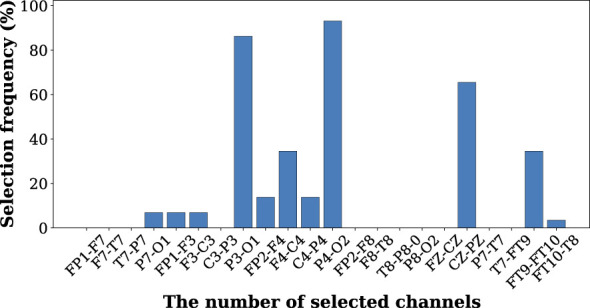
The selection frequency of each channel across the Pareto-optimal solution set.

The spatial importance of EEG channels is evaluated by calculating the selection frequency of each channel across the Pareto-optimal solution set. The selection frequency represents the proportion of Pareto solutions in which a given channel is included. Figure 2 shows the results of the distribution of the selected channels. It is observed that several channels show higher selection frequencies than others. In particular, P3-O1, P4-O2, and CZ-PZ appear the most frequently in the Pareto-optimal configurations, indicating that these channels provide the highest information for seizure classification. Furthermore, these three channels located in the postior region play important roles in cortical network activity and may capture electrophysiological alterations associated with epileptic events. In contrast, many frontal and peripheral channels exhibit low selection frequencies, suggesting that they contribute less discriminative information when both the electrode number and processing cost are constrained.

**Figure 3:**
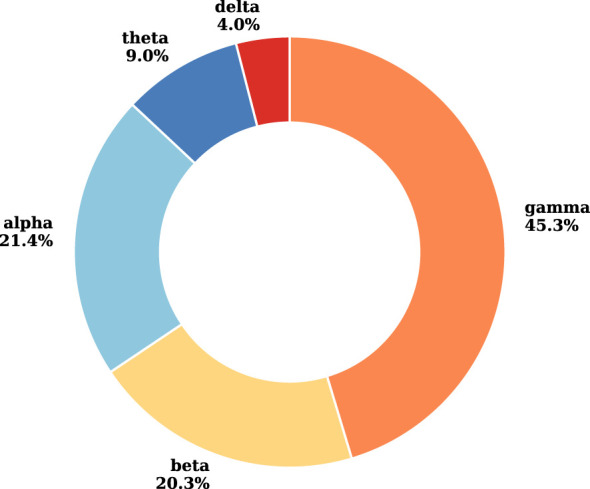
The selection proportion of each EEG frequency band across the Pareto-optimal solution set.

To further explore the spectral characteristics of the optimal configurations, we analyze the selection proportion of each EEG frequency band. For each Pareto-optimal solution, we calculate the proportion of selected channel-and-frequency-band pairs belonging to that frequency band to the total number of selected channel-and-frequency-band pairs, and then obtain the average proportion of each frequency band by averaging over all Pareto optimal solutions. The results in Figure 3 show that the gamma band accounts for the largest proportion (45.3%), followed by the alpha (21.4%) and beta bands (20.3%), whereas the theta (9.0%) and delta bands (4.0%) contribute smaller proportions. This pattern suggests that higher-frequency EEG components play key roles in the optimal seizure classification configurations. Previous studies have reported that epileptic activity may involve abnormal high-frequency oscillations (17, 18). The relatively lower representation of delta and theta bands indicates that low-frequency components may provide less discriminative information in the classification framework when computational efficiency is considered.

**Figure 4:**
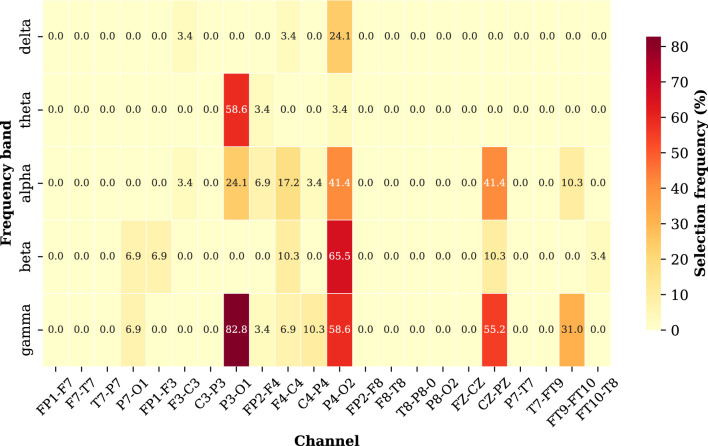
The joint selection frequency of each channel–frequency-band pair across the Pareto-optimal solutions.

To examine the interaction between spatial and spectral EEG features, we analyze the joint selection frequencies of channel-and-frequency-band pairs across the Pareto-optimal solutions and visualize them as a heatmap in Figure 4. We can observe that the combinations of P3-O1 with gamma and theta bands, P4-O2 with beta and gamma bands, CZ-PZ with alpha and gamma bands show the high selection frequencies, indicating that these spatial-spectral configurations consistently appear in high-performing solutions and capturing relevant EEG characteristics associated with the seizure activity. Overall, these conclusions highlight the importance of posterior and central EEG regions interacting with higher-frequency oscillations in seizure classification. The findings also demonstrate that the proposed framework can identify informative spatial-spectral configurations for accurate and efficient seizure classification.

### 3.3 Performance comparison between SA-NSGA-II and NSGA-II

To evaluate the effectiveness of the proposed SA-NSGA-II algorithm, we compare it with the conventional NSGA-II using two widely used performance metrics: the hypervolume (HV) and the inverted generational distance (IGD). HV measures the dominated volume of the obtained Pareto front and reflects convergence and diversity, and IGD evaluates the distance between the obtained solutions and the reference Pareto front.

**Figure 5:**
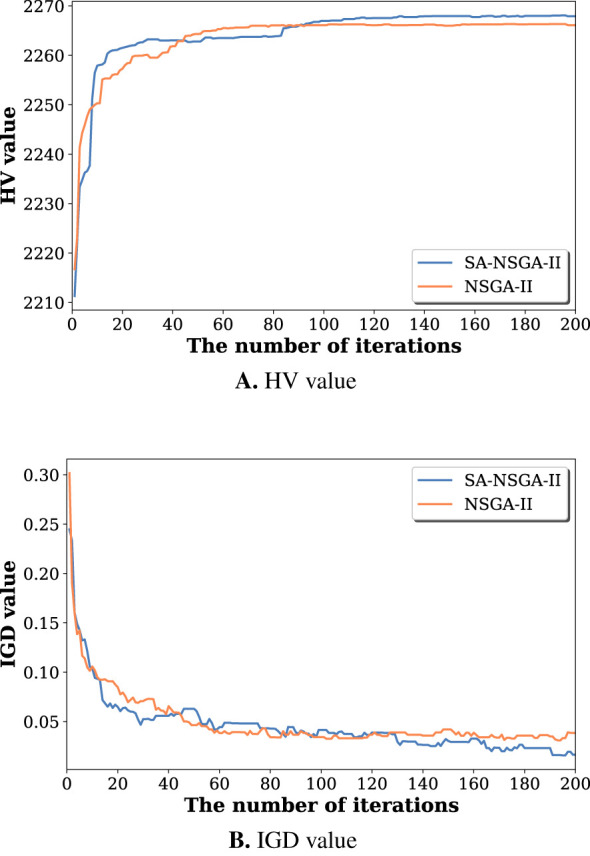
Performance comparison between SA-NSGA-II and NSGA-II. **(A)** The HV value with respect to the number of iterations. **(B)** The IGD value with respect to the number of iterations.

Figure 5 shows the HV and IGD values with respect to the number of iterations. As the optimization proceeds, both methods show performance improvement. However, SA-NSGA-II achieves higher HV values and lower IGD values than the standard NSGA-II when the optimization terminates. The results suggest that incorporating the two structure-aware mechanisms into the evolutionary search improves optimization efficiency and solution quality. Consequently, the proposed SA-NSGA-II framework is more effective in identifying EEG channel-and-frequency-band configurations for seizure classification.

## 4 Discussion

### 4.1 Positioning within EEG-based seizure classification research

The rapid development of machine learning and artificial intelligence has led to a paradigm shift in the field of seizure classification, with automatic methods gradually replacing traditional clinical examinations (19, 20). Automatic seizure classification methods based on EEG provide numerous advantages, including higher reproducibility, less reliance on experts, and efficient processing of long-term recordings (5, 21–23). Therefore, such methods have become mainstream in seizure classification. To improve classification performance, current studies have focused on addressing challenging problems such as poor model generalization due to patient variability, severe imbalance between normal and non-stationary signals. Ahmad et al. (24) proposed a hybrid deep learning method to address the high computational complexity and imbalanced medical data. This method uses oversampling techniques to balance the data and integrates a convolutional neural network with a bidirectional long short-term memory network to extract series information. Liu et al. (25) designed a group cosine convolutional neural network to address the issue that the outcomes of deep learning-based classification models often lack spatial information related to EEG channels. Tian and Zhang (26) utilized graph correlation analysis for epilepsy classification. This method detects abnormal channels and segments through inter-channel correlation analysis. To represent the temporal dynamics of epileptic brain networks, Liao et al. (27) introduced an effective seizure prediction model that integrated consistent epileptic network processes into a higher-order latent space. Existing models based on a single dataset often have insufficient robustness in clinical practice. To this end, Chen et al. (28) proposed a multi-source data adaptive framework for multicenter epileptic seizure classification. Ren et al. (29) designed a novel hybrid deep learning framework called CNN-Autoformer to address noise and the complex spatiotemporal dynamics of non-stationary EEG signals. These studies significantly improve seizure classification performance in offline assessment environments (30, 31).

Despite the improvement in prediction accuracy, real-time application remains a major bottleneck for clinical deployment. Many existing methods rely on high-density EEG recordings, complex feature extraction processes, or computationally intensive models, which limits the applicability to wearable devices, bedside monitoring systems, or long-term continuous observation. Practical seizure classification systems must operate within the resource constraints. To this end, our study explicitly incorporates real-time factors into the modeling framework. Specifically, we jointly optimize EEG channels and frequency bands. This method directly balances classification performance with signal acquisition/processing costs. Our experimental results demonstrate that reliable seizure classification can be achieved using a significantly reduced number of channels and frequency bands, which supports the deployment of automatic classification systems in real-time and resource-constrained environments.

### 4.2 Clinical implications for real-time and resource-constrained EEG monitoring

Real-time applications and resource constraints are major obstacles to the practical deployment of automatic EEG-based classification systems (32). In practice, classification systems must balance predictive accuracy with limitations of computational power and energy consumption. To improve efficiency, some studies have focused on developing lightweight machine learning models to reduce computational complexity while maintaining acceptable performance. For example, Zanetti et al. (33) employed a strategy to reduce model size and a multi-batch data processing scheme to optimize RAM usage in order to implement cognitive workload monitoring on wearable devices. Qiu et al. (3) designed a lightweight deep learning model for real-time seizure classification which utilized one-dimensional convolution, global average pooling, and kernel-wise pruning to compress the model. Jayanthi and Sivakumar (31) presented a novel machine learning network which combined quantum wavelet transform and quantum Fourier transform with dimensionality reduction methods to realize real-time seizure classification. Lou et al. (34) introduced an adaptive integrate-and-fire spiking neuron model which could be applied efficiently in large-scale or real-time seizure classification. Furthermore, Palumbo et al. (35) focused on system-level solution and designed a wearable device-based system for real-time EEG-based monitoring. Lee et al. (36) provided an algorithmic framework for real-time epilepsy classification, a dedicated coprocessor chip for implementing the framework, and a custom interface from both the hardware and software perspectives. A multi-channel EEG signal processing method that run on microcontrollers with limited memory and processing power was designed by Dan et al. (37). Ra et al. (38) introduced an EEG channel selection method based on permutation entropy to identify information-rich channels, which achieved an optimal trade-off between computational efficiency and classification accuracy.

This study addresses the efficiency issue from the perspective of signal resource allocation. By jointly selecting optimal channels and frequency bands, our framework directly reduces the number of sensors and the amount of signal processing. This method reduces the computational burden at the signal level before the model inference begins, which provides a promising pathway for real-time EEG deployment.

### 4.3 Comparison with prior EEG channel selection and optimization methods

EEG signals can reflect the state of brain neural activity and are widely used in epilepsy seizure recognition tasks. However, high-density EEG recordings often contain significant redundancy. Selecting information-rich channels helps reduce computational complexity, suppress noise interference, and improve model generalization (38, 39). Consequently, recent research has focused on selecting informative channels. For example, Handiru and Prasad (40) proposed an iterative multi-objective optimization approach for channel selection to alleviate computational burden in motor imagery decoding. Wang et al. (41) utilized normalized mutual information to identify the optimal EEG channels. Moctezuma and Molinas (8) and Jana and Mukherjee (9) selected optimal channels to maximize classification accuracy while reducing the number of channels required for seizure classification. Shen et al. (42) chose optimal channels for depression classification based on kernel-target alignment. Aljalal et al. (43) focused on selecting optimal channels and features for detecting mild cognitive impairment. Alkhrijah et al. (44) introduced a surrogate channel approach that not only reduced the channel usage but also retained the most informative ones contributing significantly to epileptic seizure prediction. Awan et al. (45) designed a dynamic channel selection method that assigned learnable weights to channels. This allows the model to adaptively prioritize channels based on their relevance to the classification objective.

These studies have alleviated the issue of spatial redundancy to some extent. However, most existing approaches focus only on channel-level optimization and overlook differences in the contribution of various frequency bands to seizure classification. As a result, such methods may introduce additional computational overhead associated with spectral analysis, limiting their applicability in real-time scenarios. Based on traditional channel selection, our method jointly optimizes the channel-and-frequency-band selection to explicitly address spectral redundancy and enabling finer-grained control over signal resources.

In essence, EEG channel selection is a black-box optimization problem; therefore, evolutionary algorithms are widely used in this research domain (15), with NSGA-II being one of the most commonly used optimization algorithms (8, 9, 43). However, the optimization model in this study introduces structural coupling constraints between channels and frequency bands, rendering the search space both restricted and complex. To overcome this challenge, we propose a structure-aware NSGA-II that enhances search efficiency and solution feasibility through a structure-preserving initialization strategy and a structure-guided search mechanism. Experimental results demonstrate that the proposed algorithm outperforms standard NSGA-II in terms of both solution quality and convergence speed.

### 4.4 Limitations and future work

This paper has some limitations and future work to explore. First, the proposed framework is evaluated only on the CHB-MIT scalp EEG database. While this dataset is widely used in epilepsy research, the patient size and recording conditions remain limited. Therefore, we need to validate the generalizability of the method on larger and more diverse clinical datasets. Second, this study focuses on identifying globally informative EEG channel-and-frequency-band configurations without considering patient specificity. Future work could explore personalized selection strategies for individual patients. Finally, the proposed method needs to be validated in practical wearable or bedside EEG monitoring systems.

## Data Availability

Publicly available datasets were analyzed in this study. This data can be found here: https://archive.physionet.org/physiobank/database/chbmit/.
